# Resting State Functional Connectivity of the Thalamus in North Korean Refugees with and without Posttraumatic Stress Disorder

**DOI:** 10.1038/s41598-020-59815-5

**Published:** 2020-02-21

**Authors:** Sehyun Jeon, Yu Jin Lee, Inkyung Park, Nambeom Kim, Soohyun Kim, Jin Yong Jun, So Young Yoo, So Hee Lee, Seog Ju Kim

**Affiliations:** 1 Department of Psychiatry, Sungkyunkwan University School of Medicine, Samsung Medical Center, Seoul, Republic of Korea; 2 Department of Psychiatry and Center for Sleep and Chronobiology, Seoul National University College of Medicine and Seoul National University Hospital, Seoul, Republic of Korea; 3 Department of Biomedical Engineering Research Center, Gachon University, Incheon, Republic of Korea; 4 Department of Neurology, Gangneung Asan Hospital, Gangwon-do, Republic of Korea; 5 National Center for Mental Health, Seoul, Republic of Korea; 6 Department of Psychiatry, National Medical Center, Seoul, Republic of Korea

**Keywords:** Stress and resilience, Post-traumatic stress disorder

## Abstract

In posttraumatic stress disorder (PTSD), functional connectivity (FC) between the thalamus and other brain areas has yet to be comprehensively investigated. The present study explored resting state FC (rsFC) of thalamus and its associations with trauma-related features. The included subjects were North Korean refugees with PTSD (n = 23), trauma-exposed North Korean refugees without PTSD (trauma-exposed control [TEC] group, n = 22), and South Korean healthy controls (HCs) without traumatic experiences (HC group, n = 40). All participants underwent psychiatric evaluation and functional magnetic resonance imaging (fMRI) procedures using the bilateral thalamus as seeds. In the TEC group, the negative rsFC between each thalamus and its contralateral postcentral cortex was stronger relative to the PTSD and HC groups, while positive rsFC between the left thalamus and left precentral cortex was stronger in the HC group compared to the PTSD and TEC groups. Thalamo-postcentral rsFC was positively correlated with the CAPS total score in the TEC group, and with the number of traumatic experiences in the PTSD group. The present study identified the difference of thalamic rsFC alterations among traumatized refugees and HCs. Negative rsFC between the thalamus and somatosensory cortices might be compensatory changes after multiple traumatic events in refugees.

## Introduction

Posttraumatic stress disorder (PTSD) can be understood as dysfunction in the amygdala, medial prefrontal cortex, hippocampus, and anterior cingulate cortex^1–3^. In addition to these brain regions, the thalamus may also be involved in the pathophysiology of PTSD. Lanius *et al*.^4^ reported that PTSD patients show less activation in the thalamus in response to script-driven imagery, and suggested that this may be related to dissociation induced by trauma recall. The same research group observed lower thalamic activation in PTSD participants during negative emotional states, which may reflect general dysregulation of affect in individuals with PTSD^5^. Findings from a single photon emission computed tomography (SPECT) study suggest that reduced thalamic blood flow of PTSD patients may be a strategy to attenuate the re-experience^6^.

Despite evidence showing thalamic involvement in the neural circuits underlying PTSD, few studies have investigated the interactions between the thalamus and other brain regions in PTSD. Recently, resting state functional connectivity (rsFC) has gained increasing attention because it reflects the intrinsic properties of the brain and provides insight into the interactions of brain regions in the absence of confounding by task-related influences^2,7^. Diffusion tensor imaging analyses, electrophysiological assessments, and perfusion studies have also shown that PTSD patients exhibit alterations in the thalamocortical pathway^8–11^. In addition, the thalamic hyper-connectivity has been reported to be associated with early life stress and anxiety in a trans-diagnostic study^12^. However, only a single study has investigated rsFC between the thalamus and cortical regions in PTSD using functional magnetic resonance imaging (fMRI)^13^.

The primary subjects in the present study were North Korean refugees who experienced repeated diverse trauma^14^, which allows for an investigation of the effects of accumulative traumatic experiences. The present study included two different control groups: traumatized refugees without PTSD and healthy controls (HCs) without trauma exposure. Comparisons of these three groups may help not only to elucidate the altered neural circuitry underlying PTSD symptoms, but also to identify the neurobiological characteristics of resilient victims who did not develop PTSD even after multiple traumatic events.

Based on associations between the thalamus and PTSD as reported in previous studies, the present study aimed to investigate rsFC between the thalamus and cortical brain regions, as well as the relationships of this rsFC with PTSD-related characteristics. We hypothesized that there would be significant differences in thalamic rsFC among North Korean refugees with PTSD, traumatized North Korean refugees without PTSD, and HCs. Additionally, we hypothesized that the clinical aspects of PTSD such as PTSD severity would be associated with rsFC of the thalamus.

## Results

### Demographic and clinical characteristics

Table 1 shows the demographic and clinical characteristics of the participants. There were no significant differences in sex and years of education. The mean age of the PTSD participants was significantly higher (40.87 ± 12.12 years) than that of the HCs (34.80 ± 11.65 years) and trauma-exposed controls (TECs) (31.55 ± 8.22 years; p = 0.018). All participants in the two refugee groups had had at least one traumatic experience, but the number of experienced traumas was significantly higher in the PTSD group than in the TEC group (6.26 ± 3.17 vs. 3.50 ± 2.28; p = 0.002). As expected, the PTSD group had significantly higher total CAPS scores for both current (41.22 ± 27.07 vs. 5.55 ± 9.86; p < 0.001) and lifetime (68.96 ± 19.34 vs. 12.00 ± 11.98; p < 0.001) PTSD symptoms than the TEC group.

**Table 1:** Demographic and clinical characteristics of the participants.

Variable	PTSD group (n = 23)		Trauma-exposed controls (n = 22)		Healthy controls (n = 40)		Group differences	
	Mean or N	SD or %	Mean or N	SD or %	Mean or N	SD or %	F/T/χ^2^	p-value
Age (years)	40.87	12.12	31.55	8.22	34.80	11.65	4.30	0.018*
Sex (female)	20	87.0	15	69.2	30	75.0	2.29	0.318
Duration of defection (months)	115.65	62.91	113.50	65.74	—	—	0.11	0.911
Length of stay in transit countries (months)	45.86	45.58	46.09	58.21	—	—	−0.02	0.988
Duration of habitation in South Korea (months)	61.74	29.31	68.09	36.94	—	—	−0.64	0.525
Number of traumatic experiences	6.26	3.17	3.50	2.28	—	—	3.34	0.002*
CAPS total, current	41.22	27.07	5.55	9.86	—	—	5.92	<0.001*
Re-experience	8.57	7.31	1.41	3.15	—	—	4.29	<0.001*
Avoidance	14.13	10.06	1.68	3.24	—	—	5.64	<0.001*
Hyperarousal	18.52	17.10	2.45	4.99	—	—	4.32	<0.001*
CAPS total, lifetime	68.96	19.34	12.00	11.98	—	—	11.93	<0.001*
Re-experience	22.48	6.62	4.82	4.71	—	—	10.27	<0.001*
Avoidance	21.39	10.24	3.95	5.16	—	—	7.26	<0.001*
Hyperarousal	25.09	7.22	3.23	4.10	—	—	12.41	<0.001*
BDI	24.65	14.95	8.00	7.97	5.15	5.64	29.87	<0.001*

SD, standard deviation; PTSD, posttraumatic stress disorder; CAPS, Clinician-administered PTSD scale; BDI, Beck Depression Inventory.

*p < 0.05.

Of the 22 TEC participants, 17 had a CAPS score greater than zero, which indicates the presence of subthreshold PTSD symptoms. Participants in the HC group did not have any trauma experiences; therefore, the current and lifetime CAPS total scores were zero for all participants. The PTSD and TEC groups did not significantly differ in duration of defection, length of stay in transit countries, or duration of habitation in South Korea. The PTSD group had a significantly higher BDI score than the HC group (p < 0.001), whereas the TEC group did not (p = 0.117).

### Group differences in rsFC

**Figure 1:**
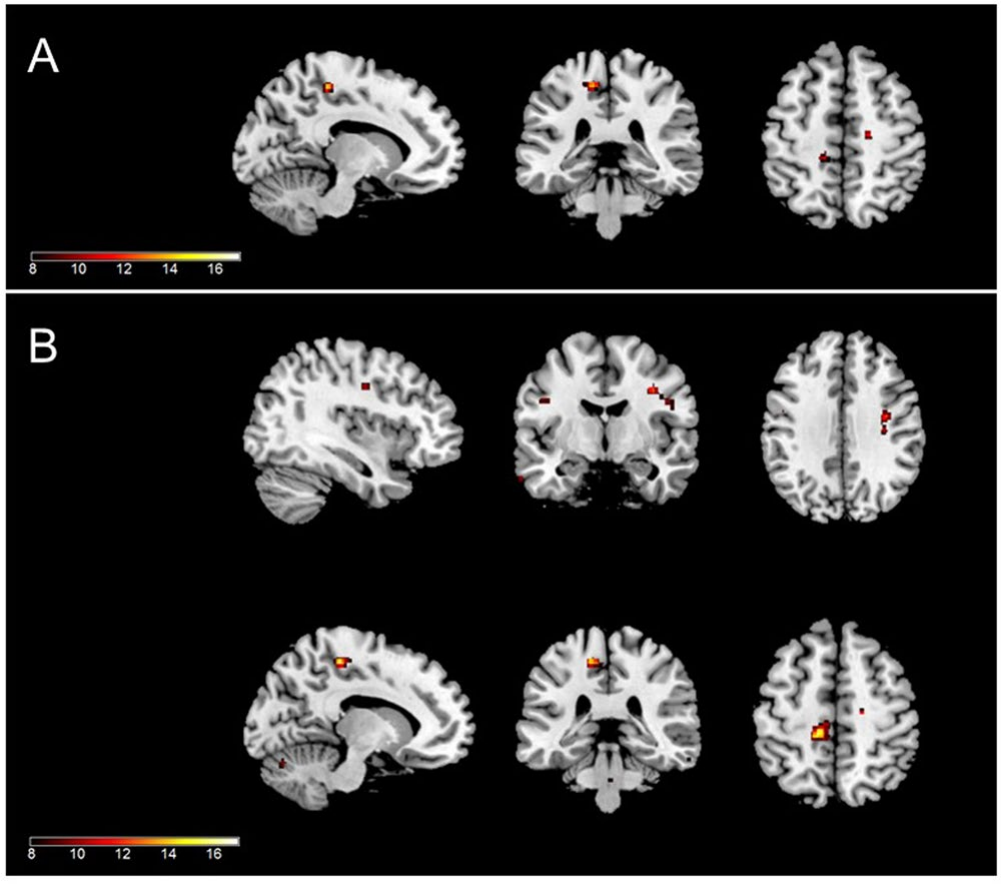
Brain areas showing differences in resting state functional connectivity (rsFC) with each thalamus among the posttraumatic stress disorder (PTSD), trauma-exposed control (TEC), and healthy control (HC) groups; with right thalamus (**A**) and left thalamus (**B**) as the seed region. The TEC group exhibited a significantly decrease in rsFC between the right thalamus and left postcentral gyrus (**A**) and between the left thalamus and right postcentral gyrus (**B**, bottom). The rsFC between between the left thalamus and left precentral gyrus (**B**, top) was significantly increased in the HC group compared to the PTSD and TEC groups.

**Figure 2:**
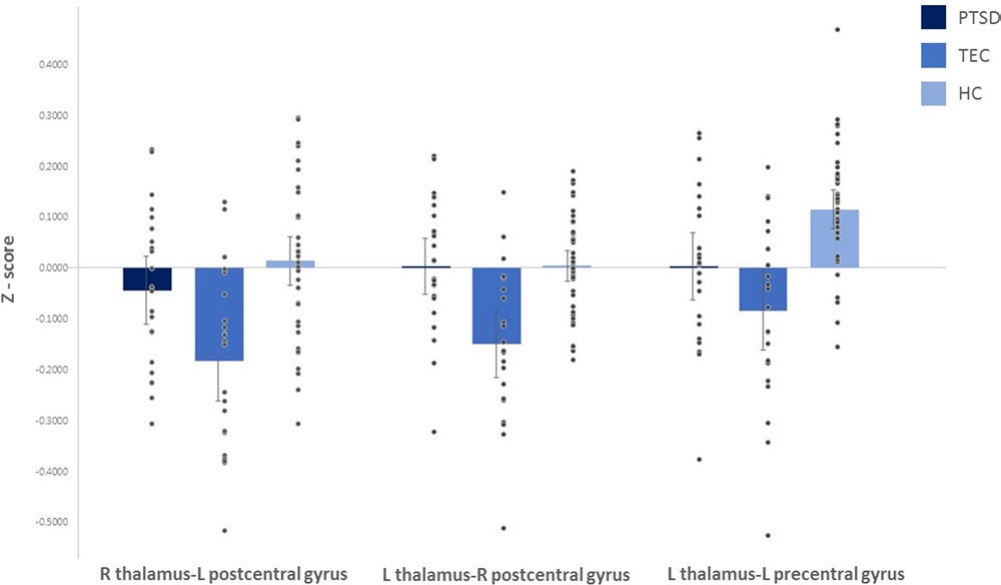
Comparison of thalamic functional connectivity strengths by cortical regions among the posttraumatic stress disorder (PTSD), trauma-exposed control (TEC), and healthy control (HC) groups. The TEC group exhibited a significant change in negative rsFC between the right thalamus and left postcentral gyrus and between the left thalamus and right postcentral gyrus, compared to the PTSD and HC groups. Meanwhile, the HC group exhibited a significant increase in positive rsFC between the left thalamus and left precentral gyrus compared to the two trauma-exposed groups. Each bar represents the mean Z-score of connectivity strength and associated standard error. L, left; R, right; PTSD.

Using the bilateral thalamus as the seed region, significantly altered rsFC relationships were observed in three brain areas (Table 2 and Fig. 1). Comparison of thalamic rsFC strengths among the PTSD, TEC, and HC groups in these areas is presented in Fig. 2. Compared to the PTSD and HC groups, the TEC group exhibited a significant increase in negative in rsFC between the right thalamus and left postcentral gyrus (Brodmann area [BA] 6; MNI coordinates x, y, and x = −48, 4, and 36; z score = 4.230; p = 0.001) and between the left thalamus and right postcentral gyrus (BA 6; MNI coordinates x, y, and x = 36, −8, and 36; z score = 4.225; p = 0.002).

**Table 2:** Differences in resting state functional connectivity in the thalamus among the PTSD, TEC, and HC groups.

Seed	Brain region	BA	One-way ANOVA Post hoc t-test	Post hoc Mean ± Standard error			MNI coordinates			Cluster size (voxels)	Peak value (z)	FWE-corrected p-value
				PTSD	TEC	HC	x	y	z			
Thalamus, R	Postcentral gyrus, L	6	PTSD, HC > TEC	−0.047 ± 0.033	−0.193 ± 0.034	0.012 ± 0.025	−48	−4	36	135	4.230	0.001*
Thalamus, L	Precentral gyrus, L	5	HC > PTSD, TEC	−0.036 ± 0.028	−0.113 ± 0.029	0.078 ± 0.021	−12	−32	54	117	4.729	0.018*
	Postcentral gyrus, R	6	PTSD, HC > TEC	0.003 ± 0.024	−0.154 ± 0.025	0.006 ± 0.018	36	−8	36	173	4.225	0.002*

BA, Brodmann area; ANOVA, analysis of variance; MNI, Montreal Neurological Institute; FWE, family-wise error rate; L, left; R, right; PTSD, post-traumatic stress disorder; HC, healthy controls; TEC, trauma-exposed controls.

*p < 0.05.

Additionally, the HC group exhibited a significantly greater positive rsFC between the left thalamus and left precentral gyrus (BA 5; MNI coordinates x, y, and x = −12, −32, and 54; z score = 4.729; p = 0.018) compared to the two trauma-exposed groups.

### Associations between thalamic rsFC and the clinical features of PTSD

To investigate the functional meaning of the abovementioned changes in regional connectivity, partial correlation analyses were performed within the PTSD group and within the TEC group, separately.

In the PTSD group, rsFC between the right thalamus and left postcentral gyrus was positively correlated with the number of traumatic experiences (r = 0.445, p = 0.043) after adjusting for age and sex; this relationship exhibited slight above the statistical significance when BDI score was also controlled for (r = 0.480, p = 0.051). Connectivity strength between the left thalamus and right postcentral gyrus also had a positive correlation with the number of traumatic experiences (r = 0.534, p = 0.013) after adjusting for age and sex; this relationship remained significant when BDI score was also controlled for (r = 0.502, p = 0.040). These correlation between the thalamo-postcentral rsFC and the number of traumatic experiences maintained its statistical significance, even when the current total CAPS score were additionally controlled for (r = 0.693, p = 0.03 for association between right thalamus and left postcentral gyrus; r = 0.682, p = 0.004 for association between left thalamus and right postcentral gyrus). No regions in the PTSD group exhibited a significant correlation between FC and CAPS scores.

Within the TEC group, connectivity strength between the right thalamus and left postcentral gyrus was positively correlated with the current CAPS total score (r = 0.544, p = 0.013) after adjusting for age and sex; this relationship remained significant when BDI score and the number of traumatic experiences were also controlled for (r = 0.561, p = 0.024). Connectivity strength between the left thalamus and right postcentral gyrus was also positively correlated with the current and lifetime CAPS total scores (r = 0.653, p = 0.002 and r = 0.465, p = 0.039, respectively) after adjusting for age and sex; these relationships remained significant when BDI score and the number of traumatic experiences were also controlled for (r = 0.658, p = 0.006 and r = 0.519, p = 0.039, respectively). There was no region of which thalamic rsFC exhibited a significant correlation with the number of traumatic experiences in this group.

The connectivity strength between the left thalamus and left precentral area was not significantly correlated with any of the clinical variables both in PTSD and TEC groups.

## Discussion

The present study demonstrated that rsFC between the thalamus and pre- and postcentral cortices significantly differed among PTSD, TEC, and HC groups, with a strict threshold using FWE multiple comparisons. The alterations in thalamo-cortical connectivity showed different associations with clinical features among the three groups. The present findings are consistent with the study hypotheses and support the notion that alterations in thalamic connectivity contribute to the pathophysiology of PTSD.

Notably, only the traumatized controls showed stronger negative rsFC between the thalamus and postcentral cortex. The thalamus serves as a relay station for incoming information from external sources to different parts of the cortex^15^, and decreased activation in somatosensory areas (i.e., postcentral cortex) is indicative of attenuated sensory processing^16^. Lower thalamo-postcentral connectivity might represent decreased sensorimotor integration^8^ or attenuated sensory responses^17^ after exposure to trauma. Because decreased thalamo-somatosensory connectivity was only observed in the TEC group, this altered connectivity may be compensatory processing aimed at repressing the somatosensory symptoms of PTSD. The TEC group may have enhanced resilience against the PTSD by reducing somatosensory responses to external sensory stimuli that are potentially related to trauma. It is also possible that the increased negative connectivity was related to neurobiological traits prior to the traumas. As Kennis *et al*.^18^ proposed that specific neural alterations may be related to resilience in traumatized controls, low rsFC between these regions may reflect an information processing style used by individuals who are resistant to the development of PTSD.

Several previous studies reported the involvement of somatosensory areas in PTSD. For example, PTSD patients exhibit increased activation in the postcentral cortex during tasks that reflect inhibitory responding^16^. Magnetoencephalography studies investigating veterans showed that PTSD patients exhibit weaker postcentral activity towards non-threatening stimuli^19^, and stronger precentral and postcentral activities during a resting state^20^. The present results are partially consistent with these findings showing stronger postcentral activity under resting conditions in PTSD patients than TECs.

The likelihood of developing PTSD depends on the interaction between individual risk factors (i.e. genetic or epigenetic risks, psychological resiliency) and traumatic load^21^, whereas cumulative trauma exposures have a clear dose-effect relationship with the symptoms of PTSD in a population with high levels of traumatic events^22–24^. However, TEC group showed the stronger thalamo-postcentral negative connectivity than PTSD group even after controlling for traumatic load. Our finding suggests that the negative thalamo-postcentral rsFC of TEC group was not due to their lower traumatic loading.

In the present study, in the PTSD group, thalamo-postcentral connectivity was correlated with the number of traumatic experiences, but not with PTSD severity. In contrast, the strength of thalamo-postcentral connectivity was correlated with PTSD severity, but not the number of traumatic experiences, in the TEC group. Although the present results could not confirm this, it is possible that the association between thalamo-postcentral connectivity and PTSD severity seen in the TEC group was due to the presence of subthreshold PTSD symptoms in these individuals. Even in the absence of definite PTSD, decreases in thalamo-somatosensory connectivity could mitigate subthreshold symptoms in traumatized individuals. However, after the onset of PTSD, reduced thalamic connectivity no longer appears to be associated with PTSD severity. In patients with PTSD, efforts to reduce thalamo-postcentral connectivity seem to work in the early phases of trauma exposure, as evidenced by the association between thalamo-postcentral connectivity and the number of traumatic experiences. However, because efforts to reduce thalamo-somatosensory connectivity repeatedly failed, it is unlikely that the strategy of compensatory thalamo-somatosensory deactivation would be maintained following repeated traumatic experiences. Without this compensation, connectivity between the thalamus and postcentral cortex may rebound as traumatic experiences accumulate.

In the present study, both refugee groups exhibited heightened thalamo-precentral negative rsFC compared to the HC group. This reduction in connectivity was correlated with both the number of traumatic experiences and PTSD severity, which suggests that it may be associated with the trauma itself and PTSD severity in traumatized people. Compared to HCs, veterans without PTSD^18^ and patients with PTSD^25^ exhibit altered FC in the precentral cortex. Because the precentral cortex is involved in motor activity, attention, and memory^26,27^, altered connectivity in this region in PTSD might be related to abnormal working memory^25^. Further research with trauma victims will be necessary to clarify the functional meaning of thalamo-precentral connectivity.

As mentioned above, only a single study has explored rsFC of thalamus in earthquake-induced PTSD^13^. These authors reported that PTSD patients show lower thalamic connectivity with the medial frontal and anterior cingulate cortices, but higher thalamic connectivity with the precuneus and inferior frontal, middle frontal, and inferior parietal cortices, compared to survivors without PTSD. Although both studies implicate thalamo-cortical connectivity during a resting state in PTSD, the patterns of thalamic connectivity differ from each other. This discrepancy may be due to differences in the characteristics of the long-term repeated trauma of refugees in the present study versus the short-term isolated trauma in the previous study. Absence of control group without trauma exposure in the previous study also limits to determine whether the observed connectivity alterations in PTSD were change in PTSD patients or in control patients.

The present study was specifically conducted in refugees. The traumas are distinctive in that refugees suffer extraordinary repeated life events such as torture, incarceration, extreme starvation, etc.^14^ On the other hand, refugees can be considered to have strong coping mechanisms for extreme adversity^28^ and likely possess enormous courage that allows them to escape from unjustifiable situations in the first instance^29^. Therefore, the rsFC alterations in the refugees in the present study may be due to the unique nature of their trauma or their strength in terms of overcoming trauma. Thus, a replication of these findings with other trauma populations will be necessary to generalize the present results.

This study had several limitations. First, the difference in mean age among the groups exists. To reduce the potential impact of age, all statistical and imaging analyses were adjusted for age. Second, the recruitment of South Korean citizens as an HC group could have affected the findings. However, South and North Koreans share the same genetic background, language, and history; thus, South Koreans were deemed suitable HCs. Third, the present study employed a cross-sectional design and, as a result, causal relationships could not be determined. Finally, the study relied on retrospective reports of past traumatic experiences, which could have been affected by recall bias.

The present study was the first to report altered thalamic connectivity in traumatized refugees during a resting state. Notably, the trauma-exposed refugees who did not develop PTSD showed an enhanced negative rsFC between the thalamus and somatosensory cortex; this was associated with the severity of trauma or PTSD. This novel finding suggests that the decreased connectivity between the thalamus and postcentral cortex may be a compensatory mechanism aimed at diminishing PTSD symptoms after trauma, or a protective factor that decreases the likelihood of developing PTSD.

## Methods

### Participants

North Korean refugees who defected to South Korea were recruited for potential PTSD group or TEC group. Native South Korean residents who had not been exposed to trauma were recruited as HC participants. All participants were recruited via advertisements from 2013 to 2017. The exclusion criteria were as follows: history of a serious head injury, neurological disorders, serious untreated medical illness, any neurodevelopmental disorder, any metal or electronic device inside the body, claustrophobia, pregnancy, and/or being unable to lie still for more than one hour for any reason. Eighty-five participants were initially included, among which six participants were additionally excluded due to the detected artifacts of their MRI images. Ultimately, the study included 45 North Korean refugees and 40 South Korean residents; of the refugees, 23 were identified as having lifetime PTSD based on DSM-IV-TR criteria^30^ and the remaining 22 had been exposed to at least a single traumatic event but did not meet the criteria for PTSD.

All procedures in this study was performed in accordance with the Declaration of Helsinki regarding the ethical principles for medical research involving human subjects. This study was approved by the Institutional Review Board of Seoul National University Hospital and all participants voluntarily provided written informed consent following a detailed explanation of the study procedures.

### Clinical evaluation

The Korean Version of the Structured Clinical Interview Schedule for DSM-IV Axis I Disorders (SCID)^31^ was administered to all participants to determine PTSD diagnoses. Additionally, the Beck Depression Inventory (BDI)^32^ was administered to assess the severity of depression, which could affect the clinical manifestations of PTSD.

To evaluate the PTSD-related phenomena, an additional clinical interview was conducted with all refugee subjects. The current and lifetime severity for three symptom categories (re-experience, avoidance, and arousal) were measured using the Clinician-administered PTSD scale (CAPS)^33^; total scores for the current and lifetime CAPS were used in the final analyses. The types and numbers of traumas experienced were identified using the Trauma Exposure Check List for North Korean Refugees^14^, which assesses direct and indirect exposure to traumas commonly experienced during defection or residency in North Korea including torture, human trafficking, arrest or incarceration, witnessing public executions, and life-threatening starvation or cold. Period of time after defection, length of stay in transit countries, and duration of habitation in South Korea were also assessed. All evaluations were performed by trained psychiatrists or clinical psychologists.

### fMRI procedures

Functional and structural brain MRI scans were acquired on a 3T MRI system (Trio Tim, Siemens; Erlangen, Germany) with a 12-channel birdcage head coil. T1-weighted anatomical images were obtained using a 3D magnetization-prepared rapid gradient echo (3D MPRAGE) sequence with the following parameters: TR = 1,670 ms, TE = 1.89 ms, TI = 900 ms, flip angle = 9°, slice thickness = 1.0 mm, in-plane resolution = 1.0 × 1.0 mm^2^, field of view = 250 mm, and matrix size = 256 × 256. Resting state fMRI images were obtained using a T2*-weighted, echo-planar imaging sequence with the following parameters: TR = 3,500 ms, TE = 30 ms, flip angle = 90°, slice thickness = 3.5 mm, in-plane resolution = 1.9 × 1.9 mm^2^, field of view = 240 mm, and matrix size = 128 × 128. The total duration of the scanning was approximately 30 min. Each acquired images underwent visual inspection for gross motion artifacts produced by head movements of the participants during the scans, and for susceptibility artifacts due to the metallic implants. Then, the manually-identified contaminated data were removed.

The pre-processing of fMRI data was performed using SPM12 (Wellcome Trust Centre for Neuroimaging; London, UK) with the following procedures applied to each participant prior to statistical analysis. First, the origin coordinates of the structural and functional MRI images were set to the anterior commissure prior to spatial preprocessing. Then, the fMRI images were realigned to the first image to correct for head motion; the slice-timing was also corrected. Next, the fMRI images were co-registered with the anatomical images and spatially normalized to the Montreal Neurological Institute (MNI) space using a transformation matrix derived from the T1 image segmentation. After normalization, the fMRI images were spatially smoothed with a Gaussian kernel with full-width at half-maximum of 6 mm. Finally, the obtained fMRI images were visually inspected to confirm data suitability.

The CONN functional connectivity toolbox v16b^34^ was used to perform the FC analysis of the resting state fMRI data. All data were band-pass filtered (0.008–0.09 Hz) and Artifact Detection Tool (ART)-based scrubbing was performed to detect outliers. Denoising procedures to remove physiological and other spurious noise sources in the blood oxygenation level-dependent (BOLD) signal were implemented in the anatomical component-based noise correction (CompCor) strategy of CONN^35^; six motion parameters (3 rotations and 3 translations) obtained from the pre-processing procedure were also removed. Seed regions-of-interest (ROIs) in the bilateral thalamus were predefined based on the Harvard-Oxford atlas (FSL, [fMRIB, Oxford, UK])^36^ and a seed-to-voxel analysis was performed to identify functional correlations between the seed regions and other cerebral regions. For each participant, the mean BOLD signal time series was extracted for each seed region and then correlated with the BOLD signal time series of all other voxels in the brain. To apply a group-level analysis, Pearson’s correlation coefficients were converted to z-scores using Fisher’s r-to-z transformation. All group-level analyses of the PTSD, TEC, and HC groups were carried out using analysis of covariance (ANCOVA) controlling for sex and age. The reported results of the seed-to-voxel correlation analyses were thresholded at a corrected family-wise error (FWE) rate of p < 0.05 and an uncorrected peak level of p < 0.001 to control for false positives.

### Statistical analyses

ANOVAs and independent t-tests were conducted to compare the demographic and clinical variables. To examine differences among the three groups in FC using z-FC maps of the bilateral thalamus, ANOVA and post-hoc t-tests were performed. Partial correlation analyses were performed to identify associations between FC strength (mean z-value) and the clinical variables for each refugee groups. All statistical analyses were performed using IBM SPSS Statistics (ver. 24.0; IBM Corp., Armonk, NY, USA) and p-values < 0.05 were considered to indicate statistical significance.
